# Toll-like receptor 9 promoter polymorphism is associated with decreased risk of Alzheimer’s disease in Han Chinese

**DOI:** 10.1186/1742-2094-10-101

**Published:** 2013-08-20

**Authors:** Ying-Li Wang, Meng-Shan Tan, Jin-Tai Yu, Wei Zhang, Nan Hu, Hui-Fu Wang, Teng Jiang, Lan Tan

**Affiliations:** 1 Department of Neurology, Qingdao Municipal Hospital, School of Medicine, Qingdao University, Qingdao, China; 2 Department of Neurology, Qingdao Municipal Hospital, College of Medicine and Pharmaceutics, Ocean University of China, Qingdao, China; 3 Department of Neurology, Qingdao Municipal Hospital, Nanjing Medical University, Qingdao, China

**Keywords:** Alzheimer’s disease, Polymorphisms, TLR9, rs187084, Expression, Association study

## Abstract

**Background:**

Toll-like receptors (TLRs), as major innate immune mediators, may be involved in clearance of cerebral amyloid-β (Aβ) deposits. Recently, a novel TLR9 signaling pathway has been uncovered, which is functionally associated with the immune inflammatory response and reducing Aβ burden in Alzheimer’s disease (AD) mice. Therefore, *TLR9* might represent a reasonable functional candidate gene for AD.

**Findings:**

Our study investigated 1,133 sporadic late-onset AD (LOAD) and 1,159 healthy controls matched for sex and age in a large Han Chinese population. One selected functional rs187084 polymorphism within the *TLR9* gene was genotyped by polymerase chain reaction-ligase detection reaction in a case–control associated study. The *TLR9* rs187084 variant homozygote GG was significantly associated with a decreased LOAD risk after adjusting for age, gender, and ApoE ϵ4 status by logistic regression analysis (*P* = 0.035). Our result showed significant evidence of the interaction of ApoE ϵ4 with rs187084. When we further stratified our data by the ApoE ϵ4 status, we detected significant differences in the genotype and allele distributions of rs187084 between LOAD patients and controls in ApoE ϵ4 carriers (*P* < 0.001, *P* = 0.003, respectively). Moreover, we examined *TLR9* expression in peripheral blood monocytes by flow cytometry, and the GG genotype of the *TLR9* rs187084 polymorphism was associated with a higher *TLR9* expression than two other genotypes in LOAD patients.

**Conclusion:**

Our findings support the hypothesis that the *TLR9* polymorphism may modify LOAD risk in the Han Chinese population.

## Background

Alzheimer’s disease (AD) is the most common form of dementia in the elderly, characterized by a slow but progressive loss of cognitive function and memory [1]. The cardinal pathological hallmark of AD is amyloid-β (Aβ) deposits in neuritic plaques and cerebral vessels [2] which are closely associated with inflammatory responses such as activated microglia in brain [3]. Increasing evidence indicates that the immune inflammatory mechanisms are not merely bystanders in neurodegeneration but powerful pathogenetic forces in AD progression [4].

Toll-like receptors (TLRs) are a family of innate immune mediators that are expressed by a variety of immune and nonimmune cells [5]. There are at least 13 distinct TLR family members known in mammals, of which the pathogen specificities of 10 (TLR1-9 and 11) have been identified [6]. Like many other receptor families, TLRs act as heterodimers to add specificity to their recognition repertoire. Among them, TLR9 is localized to the endosomal-lysosome compartment where it can recognize unmethylated cytosine-guanosine (CpG) DNA from internalized bacteria and viruses [7,8]. Unlike TLR2 and TLR4, TLR9 agonism did not aggravate Aβ-induced microglial activation in the immune response [9]. In addition, it has been demon-strated that activation of TLRs with their specific ligands markedly boost uptake of Aβ, suggesting that TLR signaling pathways are involved in clearance of cerebral Aβ deposits [10]. However, in mononuclear cells of AD patients, transcription of TLRs upon Aβ stimulation is severely depressed [11]. More importantly, recent studies have pointed out that immune stimulation targeting TLR9 could dramatically attenuate Aβ neurotoxicity and reduce Aβ levels in *in vitro* and *in vivo* AD models [12]. Meanwhile, this reduction in amyloid was associated with cognitive improvement in AD mice [13]. Therefore, *TLR9* might represent a reasonable functional candidate gene for AD.

The human *TLR9* gene is located at 3p21.3. It has been demonstrated that polymorphisms in the *TLR9* gene affect host susceptibility to a range of diseases, such as infection, immune inflammatory diseases, and cancers [14-17]. However, no studies have investigated the association between *TLR9* polymorphisms and susceptibility to AD. In our current study, a functional variant of rs187084 located in the promoter region of *TLR9*[18] was chosen. We hypothesize that the potentially functional rs187084 polymorphism may contribute to AD susceptibility. To test the hypothesis, we performed a case–control study including 1,133 LOAD cases and 1,159 healthy controls matched for sex and age in the Han Chinese population. Then, we compare the levels of *TLR9* expression in peripheral blood monocytes of LOAD patients among the different rs187084 genotypes.

## Methods

### Subjects

Our study consisted of 1,133 LOAD (age at onset ≥ 65 years) patients and 1,159 healthy controls matched for sex and age from the Department of Neurology of the Qingdao Municipal Hospital, and several other hospitals in Shandong Province. All of the above participants were unrelated Han Chinese in origin. A clinical diagnosis of probable AD was established according to the criteria of NINCDS-ADRDA [19]. No AD patient had a family history of dementia. The control group underwent neurological and medical examinations, which showed that they were free of any symptoms suggestive of cognitive decline. An informed consent to participate in this study was obtained from each subject or from a guardian, and the protocol of this study was approved by the Ethical Committee of Qingdao Municipal Hospital.

### Genotype analysis

DNA was extracted from the peripheral blood leukocytes of patients and healthy controls using the Wizard genomic DNA purification kit (#A1125; Promega, Madison, WI, USA). The polymorphism at position rs187084 on chromosome 3p21.3 was genotyped by polymerase chain reaction-ligase detection reaction (PCR-LDR) (TapMan Assay) on an ABI Prism 377 Sequence Detection System (Applied Biosystems, Foster City, CA, USA), with technical support from the Shanghai Genesky Biotechnology Company [20,21]. The primer sequences used for the PCR were: forward: 5′-CGTCTTATTCCCCTGCTGGAATG-3′; reverse: 5′-CCTCCCAGCAGCAACAATTCAT-3′. Data analysis was achieved using GeneMapper Software v4.0 (Applied Biosystems, Foster City, CA, USA). Results of the PCR-LDR method corresponded with the results of sequencing for the randomly selected DNA samples from each genotype. ApoE was also genotyped by the PCR-LDR method.

### TLR9 expression analysis by flow cytometry

Venous blood was obtained from 60 LOAD patients (20 randomly selected cases from each genotype). Whole blood cells collected in sodium heparin tubes were stained with fluorescein isothiocyanate (FITC)-conjugated anti-human CD14 (BD Biosciences, San Jose, CA, USA) to identify the monocyte population. After being washed, the cells were permeabilized, and then stained with anti-human TLR9 antibody (eBioscience, San Diego, CA, USA). The isotype-matched IgG2a antibody was used as control. After being washed again, cells were incubated with phycoerythrin-conjugated secondary antibody. Expression of TLR9 was gated and analyzed by flow cytometry (Beckman Coulter, Fullerton, CA, USA) as mean fluorescence intensity (MFI) with regard to isotype control signal. The data were calculated with Cell Quest Pro Software (Becton, Dickinson and Company, USA).

### Statistical analysis

Hardy-Weinbery equilibrium (HWE) was tested with the *χ*^2^ test to calculate genotype and allele distribution. Statistical differences between AD cases and control subjects were also tested when we stratified our data by ApoE ϵ4 status. Differences in the characteristics for our subjects were examined using the Student *t*-test or the *χ*^2^ test. Odds ratio (OR) and 95% confidence intervals (CIs) were tested using logistic regression models after adjustment for sex, age, and ApoE ϵ4 status under various genetic models that were defined as 1 (GG + GA) versus 0 (AA) for dominant, 2 (GG) versus 1 (GA) versus 0 (AA) for additive, and 1 (GG) versus 0 (GA + AA) for recessive. All statistical analyses were performed by IBM SSPS statistics 19.0 (IBM Company, USA). The statistical power of the study was calculated by STPLAN43 software. The level of significance for all statistical tests was defined as *P* < 0.05.

## Results

The distribution of the *TLR9* (rs187084) was in HWE for both AD patients and controls (*P* > 0.05). Allele and genotype frequencies of rs187084 in our control group were consistent with the CHB (Han Chinese in Beijing) genotype data in the HapMap database. Demographic and clinical characteristics of the participants are shown in Table 1. There were no differences for gender (*P* = 0.070) and age (*P* = 0.078) between AD and controls. We found significantly lower Mini-Mental State Examination (MMSE) scores in LOAD patients compared to the controls (*P* < 0.001). As expected, the ApoE ϵ4 allele frequency was also significantly different between AD patients and controls (*P* < 0.001).

**Table 1:** Demographic and clinical characteristics of the study subjects

	**AD (n = 1,133)**	**Control (n = 1,159)**	***P-*value**
Age at examination (years) (mean ± SD)	79.94 ± 8.12	74.48 ± 6.29	
Age at onset (years) (mean ± SD)	75.01 ± 8.00		0.078^a^
Gender, n (%)			
Male	464 (41.0)	518 (44.7)	0.070
Female	669 (59.0)	641 (55.3)	
MMSE (mean ± SD)	10.06 ± 3.82	28.26 ± 1.08	< 0.001
ApoE ϵ4 status, n (%)			
ApoE ϵ4 carrier	315 (27.8)	158 (13.6)	< 0.001
ApoE ϵ4 noncarrier	818 (72.2)	1,001 (86.4)	

^a^*P* value was calculated with the age of onset for late-onset AD and age at examination for controls. AD, Alzheimer’s disease; ApoE ϵ4, apolipoprotein E ϵ4; MMSE, Mini-Mental State Examination.

We evaluated single-nucleotide polymorphism (SNP) rs187084 effects under different models using logistic regression adjusting for age, gender, and ApoE ϵ4 status. The rs187084 was found to significantly decrease the risk of developing LOAD in a recessive model (OR = 0.776, 95% CI = 0.613 to 0.982), but not in dominant or additive models. Besides, an interaction between rs187084 polymorphism and ApoE was also observed in different genetic models (Table 2). When we further stratified our data according to ApoE ϵ4 status, we detected significant differences in the genotype and allele distributions of rs187084 between LOAD patients and controls in ApoE ϵ4 carriers (*P* < 0.001, *P* = 0.003, respectively); and the rs187084 minor G-allele significantly decreased the risk of LOAD (OR = 0.656, 95% CI: 0.498 to 0.865). However, no significant frequency differences between AD and controls at allelic and genotype levels were observed in the total sample or in ApoE ϵ4 noncarriers (Table 3).

**Table 2:** rs187084 polymorphism association with Alzheimer’s disease (AD) according to different genetic models

**SNP**	**Model**^**a**^	**Wald**	**OR (95% CI)**	***P***	***P *for ApoE interaction**
rs187084	D	0.490	0.940 (0.791 to 1.118)	0.484	0.264
	A	2.538	0.906 (0.801 to 1.023)	0.111	0.009
	R	4.459	0.776 (0.613 to 0.982)	0.035	< 0.001

^**a**^The logistic genetic models were defined as follows:

D, dominant model, defined as 1 (GG + GA) versus 0 (AA);

A, additive model, 2 (GG) versus 1 (GA) versus 0 (AA);

R, recessive model, 1 (GG) versus 0 (GA + AA).

*P* < 0.05 was considered significant.

ApoE, apolipoprotein E ϵ4; CI: confidence interval; OR: odds ratio; SNP, single-nucleotide polymorphism.

**Table 3:** Genotype and allele frequencies for rs187084 stratified by apolipoprotein E ϵ4 (ApoE) status

**rs187084**	**n**	**Genotype**			***P***	**Allele**		***P***	**OR (95% CI)**
		**GG (%)**	**GA (%)**	**AA (%)**		**G (%)**	**A (%)**		
AD	1,133	150 (13.2)	557 (49.2)	426 (37.6)	0.100	857 (37.8)	1,409 (62.6)	0.076	0.898 (0.798 to 1.011)
Control	1,159	190 (16.4)	556 (48.0)	413 (35.6)		936 (40.4)	1,382 (59.6)		
ApoE ϵ4 carriers									
AD	315	30 (9.5)	156 (49.5)	129 (40.1)	< 0.001	216 (34.3)	414 (65.7)	0.003	0.656 (0.498 to 0.865)
Control	158	38 (24.1)	64 (40.5)	56 (35.4)		140 (44.3)	176 (55.7)		
ApoE ϵ4 noncarriers									
AD	818	120 (14.7)	401(49.0)	297 (36.3)	0.936	641 (39.2)	995 (60.8)	0.722	0.976 (0.854 to 1.116)
Control	1,001	152 (15.2)	492 (49.2)	357 (35.7)		796 (39.8)	1,206 (60.2)		

AD, Alzheimer’s disease; ApoE ϵ4, apolipoprotein E ϵ4; CI, confidence interval; OR, odds ratio.

**Figure 1:**
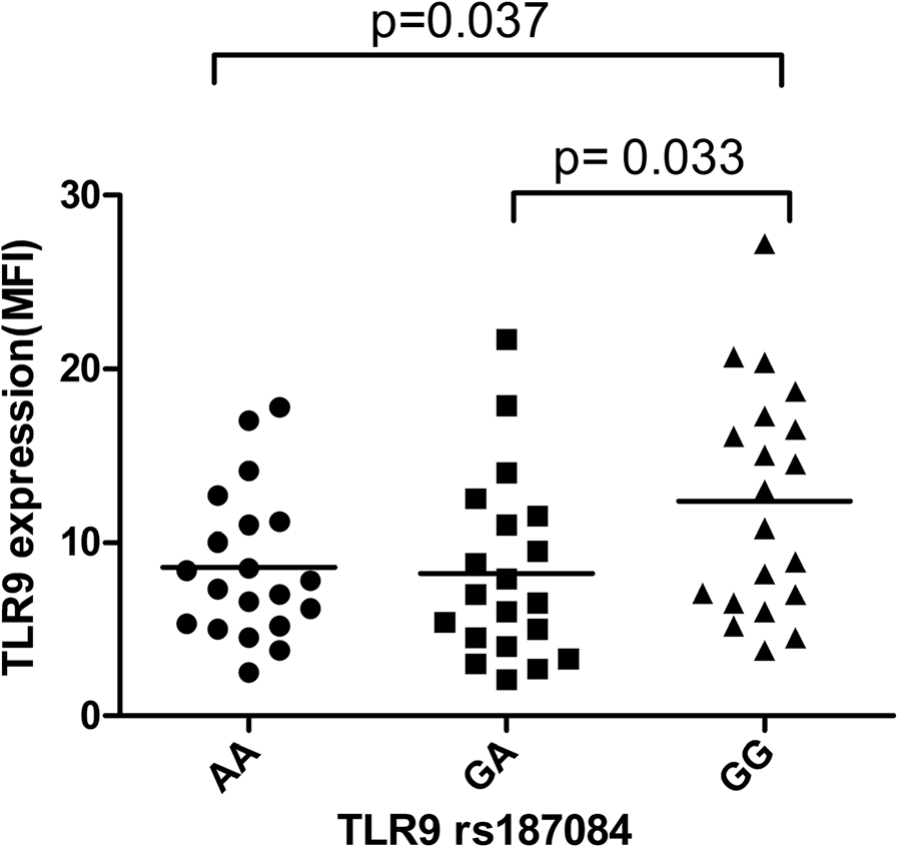
**Toll-like receptor 9 (TLR9) expression in peripheral blood monocytes of late-onset Alzheimer’s disease (LOAD) patients according to *TLR9 *genotypes.** Results are expressed as mean fluorescent intensity (MFI) units by flow cytometry. Statistically significant differences between TLR9 genotypes are indicated.

We further compared *TLR9* levels in peripheral blood monocytes of LOAD patients among the different rs187084 genotypes. Our results showed that the GG genotype of the *TLR9* rs187084 polymorphism was associated with a higher TLR9 expression than two other genotypes in LOAD patients (Figure 1).

## Discussion

To the best of our knowledge, this is the first study to evaluate whether the *TLR9* rs187084 polymorphism could influence susceptibility to LOAD in a large Han Chinese population. Logistic regression analyses showed that the rs187084 variant homozygote GG was associated with a significantly decreased LOAD risk (OR = 0.776, 95% CI = 0.613 to 0.982) in the recessive genetic model. Moreover, rs187084 showed a significant ApoE interaction. After stratifying our data by ApoE ϵ4 status, we detected significant differences in the genotype and allele distributions of rs187084 between LOAD patients and controls in ApoE ϵ4 carriers. The minor G allele was revealed to be associated with a decreased risk of LOAD. These findings indicate that the G allele of rs187084 is the protective allele against the development of LOAD. More importantly, the GG genotype of TLR9 rs187084 polymorphism was associated with a higher TLR9 expression than two other genotypes in LOAD patients.

The *TLR9* rs187084 is a potentially functional variant located in the promoter region [18] which may regulate the TLR9 transcript levels and protein function. Several studies have investigated the effect of rs187084 polymorphism on human diseases, such as rheumatoid arthritis [14], systemic lupus erythematosus [22], symptomatic malaria [23], Graves’ ophthalmopathy [24], and cervical cancer [17]. In the current study, we found that the rs187084 variant within *TLR9* affected susceptibility to LOAD in a large Han Chinese population. The minor G allele significantly decreased the risk of LOAD, and GG genotype up-regulated TLR9 expression in peripheral blood monocytes of LOAD patients, demonstrating that functionality of the rs187084 polymorphism could change TLR9 expression. Considering the key role of TLR9 in clearance of cerebral Aβ in AD progression [13], this functional variant might play a protective role in AD by increasing Aβ clearance. In-depth studies are greatly needed to confirm the functionality of the rs187084 polymorphism in the brain, and elucidate its detailed role in AD pathogenesis. In addition, only one selected SNP within the *TLR9* gene was genotyped in our study. Future comprehensive investigation of the association between other polymorphisms in *TLR9* and AD susceptibility is also warranted.

## Conclusion

Our study demonstrated an association between *TLR9* rs187084 polymorphism and LOAD risk. The G allele was revealed to be a protective factor for the LOAD in a Han Chinese population. However, it remains unknown whether it is applicable to other ethnic groups. Different populations and larger sample studies are necessary to validate our findings and further clarify the possible role of TLR9 in LOAD.

## Abbreviations

Aβ: Amyloid-β; AD: Alzheimer’s disease; APP: Amyloid precursor protein; ApoE: Apolipoprotein E; CHB: Han Chinese in Beijing; CI: Confidence interval; CNS: Central nervous system; FITC: Fluorescein isothiocyanate; HWE: Hardy-Weinbery equilibrium; LOAD: Late-onset Alzheimer’s disease; MFI: Mean fluorescence intensity; MMSE: Mini-Mental State Examination; OR: Odds ratio; PCR-LDR: Polymerase chain reaction-ligase detection reaction; TLR: Toll-like receptors.

## Competing interests

The authors declare they have no competing interests.

## Authors’ contributions

YLW, MST, and JTY were the main researchers in this study, and contributed to writing the manuscript. YLW, MST, JTY, WZ, NH, HFW, TJ, and LT were involved in collecting the blood samples and clinical data. YLW, MST, JTY and LT planned the study, wrote the protocol, were involved in the genetic and clinical aspects of data analyses, and revised the manuscript. All authors read and approved the final manuscript for publication.

## Authors’ information

Ying-Li Wang and Meng-Shan Tan are co-first authors.
